# Prospects for RNAi Therapy of COVID-19

**DOI:** 10.3389/fbioe.2020.00916

**Published:** 2020-07-30

**Authors:** Hasan Uludağ, Kylie Parent, Hamidreza Montazeri Aliabadi, Azita Haddadi

**Affiliations:** ^1^Department of Chemical and Materials Engineering, University of Alberta, Edmonton, AB, Canada; ^2^Faculty of Pharmacy and Pharmaceutical Sciences, University of Alberta, Edmonton, AB, Canada; ^3^Chapman University School of Pharmacy, Harry and Diane Rinker Health Science Campus, Irvine, CA, United States; ^4^College of Pharmacy and Nutrition, University of Saskatchewan, Saskatoon, SK, Canada

**Keywords:** drug delivery, siRNA, COVID-19, SARS-CoV-2, anti-viral drugs

## Abstract

COVID-19 caused by the SARS-CoV-2 virus is a fast emerging disease with deadly consequences. The pulmonary system and lungs in particular are most prone to damage caused by the SARS-CoV-2 infection, which leaves a destructive footprint in the lung tissue, making it incapable of conducting its respiratory functions and resulting in severe acute respiratory disease and loss of life. There were no drug treatments or vaccines approved for SARS-CoV-2 at the onset of pandemic, necessitating an urgent need to develop effective therapeutics. To this end, the innate RNA interference (RNAi) mechanism can be employed to develop front line therapies against the virus. This approach allows specific binding and silencing of therapeutic targets by using short interfering RNA (siRNA) and short hairpin RNA (shRNA) molecules. In this review, we lay out the prospect of the RNAi technology for combatting the COVID-19. We first summarize current understanding of SARS-CoV-2 virology and the host response to viral entry and duplication, with the purpose of revealing effective RNAi targets. We then summarize the past experience with nucleic acid silencers for SARS-CoV, the predecessor for current SARS-CoV-2. Efforts targeting specific protein-coding regions within the viral genome and intragenomic targets are summarized. Emphasizing non-viral delivery approaches, molecular underpinnings of design of RNAi agents are summarized with comparative analysis of various systems used in the past. Promising viral targets as well as host factors are summarized, and the possibility of modulating the immune system are presented for more effective therapies. We place special emphasis on the limitations of past studies to propel the field faster by focusing on most relevant models to translate the promising agents to a clinical setting. Given the urgency to address lung failure in COVID-19, we summarize the feasibility of delivering promising therapies by the inhalational route, with the expectation that this route will provide the most effective intervention to halt viral spread. We conclude with the authors’ perspectives on the future of RNAi therapeutics for combatting SARS-CoV-2. Since time is of the essence, a strong perspective for the path to most effective therapeutic approaches are clearly articulated by the authors.

## Brief Introduction

**FIGURE 1:**
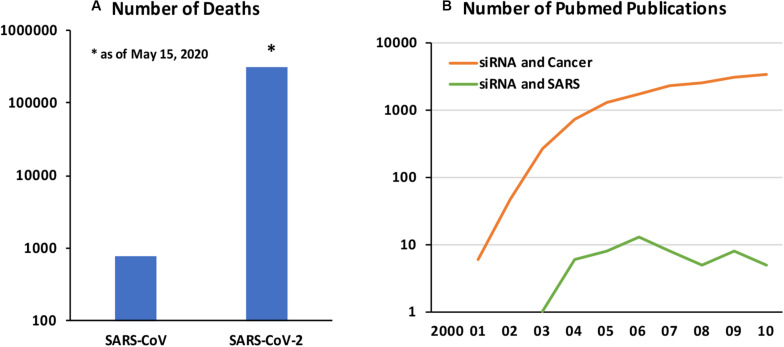
**(A)** Number of deaths associated with SARS-CoV vs SARS-CoV-2 (as of May 15, 2020). Data from World Health Organization. **(B)** Number of Pubmed publications on “siRNA and cancer” and “siRNA and SARS” between 2000 and 2010. The relative numbers of published papers in the two field is indicative of relative emphasis of deploying the newly emerging RNAi technology in cancer and SARS CoV infections.

Coronavirus Disease 2019 (COVID-19) caused by a new form of Severe Acute Respiratory Syndrome coronavirus (SARS-CoV), named SARS-CoV-2, is a fast emerging infectious disease with deadly consequences. The original SARS-CoV epidemic that spread in 2002–2004 is estimated to affect ∼8400 individuals with fatality rate of 11%, while the current epidemic has affected 3.5 M individuals with ∼305,000 loss of life as of May 15, 2020 (Figure 1A). The SARS-CoV-2 is spreading much faster but with lower mortality rates, although exact nature of the pandemic and the associated loss of life will likely be better analyzed after the passage of initial pandemic. The SARS-CoV-2 appears to enter cells via widely expressed cell-surface angiotensin−converting enzyme 2 (ACE2) and displays strong tropism against certain critical organs, in particular airways and lungs, kidneys and gastrointestinal track (Guo et al., 2008). Failure of the pulmonary and/or associated cardiovascular system is a critical factor for loss of live among the most affected patients. Other contributing factors are known to exasperate the disease and loss of life (Zheng et al., 2020). Beyond the virus-associated pathology in lung tissues, excessive pro-inflammatory response displayed against the CoV, mediated by elevated inflammatory cytokines and chemokines, lead to lung injury and acute respiratory distress syndrome (ARDS). However, other notable pathologies have been observed, including neurological complication, clotting disorders and kidney and liver failures, whose severity and consequences are just beginning to be understood.

The dynamic nature of COVID-19 makes it difficult to predict future therapeutic developments in the field but, given the significant loss of life, extreme measures are needed to minimize patient mortality. There were no effective anti-viral therapies at the present time to combat this infection. The fastest approach to therapy is to re-purpose the currently approved drugs to fight the disease, while a permanent solution could be an effective vaccine. Major efforts are placed in this direction with promising early results (Scavone et al., 2020). The re-purposed drugs could be directed to viral replication events (e.g., inhibitors of unique viral enzymes), or to host factors related to viral trafficking (e.g., inhibitors of endocytosis or viral escape). Alternatively, drugs against the adverse events could be deployed to minimize the undesirable consequences of viral host response, so that the patients are given a chance to contain the virus. The players in “cytokine response” have attracted attention in this regard (Jamilloux et al., 2020) and “neutralizing” antibodies are being actively re-positioned for this end. Like re-purposed drugs, it will take some guess work to choose the right target for neutralization. The know-how build with the original SARS-CoV endemic might guide the efforts for therapeutic development. Early data is emerging on the similarities between the SARS-CoV and SARS-CoV-2 (Yan et al., 2020), but differences are bound to take longer to be revealed. The inherent assumption is that the similarities out-weight the differences between the two CoVs and that effective therapeutics are likely to emerge from the past knowledge on CoV infections.

Developing new small molecule drugs may be challenging, including the time and effort required for drug development, in case current “re-purposed” drugs are proven ineffective. A promising approach to develop a more specific anti-viral therapy could be based on endogenous RNA interference (RNAi) mechanism whose physiological goal is to regulate protein synthesis events. RNAi has been adopted for therapy by silencing desired genes based on blockage and degradation of corresponding mRNAs. RNAi can be implemented with synthetic short interfering RNAs (siRNAs; 19–27 nucleotide long double-stranded RNAs), or *in situ* production of short hairpin RNAs (shRNAs) through typically plasmid DNA (pDNA)-based expression vectors. While the latter relies on nuclear targeting for efficient expression, siRNAs can be delivered to cytoplasmic space to engage the RNA-induced silencing complex (RISC) directly with minimal processing by host cells. Silencing a wide range of targets with RNAi are being effectively implemented at will, so that a broad therapy platform could be envisioned in this pursuit. The exciting possibilities with RNAi was recently (2018) confirmed with the FDA-approval of the first siRNA based drug (Patisiran by Alnylam) to treat the nerve damage caused by the rare disease hereditary transthyretin-mediated amyloidosis (hATTR) in adults. Developing RNAi based drugs for SARS-CoV-2 will be a lengthier process than the re-purposed, already approved drugs but it is likely to offer more specific therapies. Past attempts to control SARS-CoV infections using RNAi may guide the efforts in the current pandemic. Unfortunately, due to relatively small cases associated with SARS-CoV and especially being localized to the eastern hemisphere, not so much attention was paid to using RNAi for management of the disease (Figure 1B); from our analysis of Pubmed publications, deployment of RNAi in SARS remained only at a fraction of the cancer therapy.

In this review article, we first present a concise summary of the known infection mechanism by SARS-CoV, assuming that the current SARS-CoV-2 follows a similar pattern of cellular entry, trafficking and replication. The emphasis is to reveal possible RNAi targets rather than providing a complete picture of the associated events. We refer the reader to other sources in this Research Theme for a more comprehensive analysis of cellular entry mechanisms. We then review the available literature on the use of RNAi for understanding and control of CoV infection. We briefly outline the possible targets, industrial activity and academic efforts, with special emphasis on the critical aspects of the technology for clinical translation including drug delivery issues. We additionally analyze the feasibility of employing RNAi to control the pathogenic “cytokine storm” and finish with a review of inhalational technology that can be applied to nucleic acid therapies. Others have reviewed the RNAi approach to CoV treatment (Wu and Chan, 2006) and we refer the reader to these articles for a complementary view on the potential of RNAi mechanism for control of CoV infections.

## Mechanism of Cell Entry and Infection for SARS-CoV-2

**FIGURE 2:**
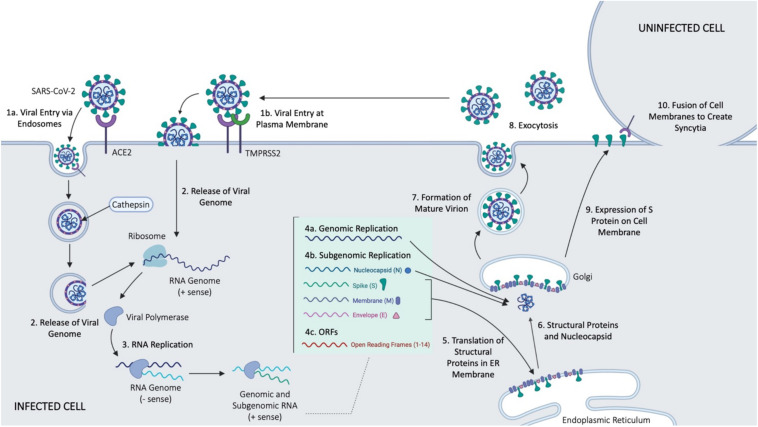
Main events in cellular entry and trafficking of CoV. Some of the outlined steps were inferred form the SARS-CoV infection of host cells. Figure courtesy of BioRender.

SARS-CoV-2 is a betacoronavirus of the family *Coronaviridae* (Gordon et al., 2020). It is an enveloped, positive-sense, single-stranded RNA virus with a genome just under 30 kb (Gordon et al., 2020). SARS-CoV-2 cell entry depends on the surface glycoprotein, or spike (S) protein common to all CoVs (Walls et al., 2020; Figure 2). The S protein is comprised of two functional subunits, S_1_ and S_2_, which mediate host-cell binding and viral entry, respectively (Zhang et al., 2020a). The S_1_ subunit contains the receptor-binding domain in its ectodomain (Musarrat et al., 2020), allowing for binding of the virus to host cell membrane. Once bound, the spike protein is cleaved by host proteases at the S_1_/S_2_ boundary and S2′ site located downstream of the S1/S2 proteolytic cleavage (Musarrat et al., 2020), priming the S_2_ fusion machinery for fusion of the viral and host cell membranes (Zhang et al., 2020a). The latter involves formation of a six-helix bundle fusion core by two heptad repeats (HR1 and HR2 domains) found in each S monomer (Musarrat et al., 2020). The bundle fusion core forms the initial pore in the membrane and ultimately leads to membrane fusion (Musarrat et al., 2020). Cellular entry of SARS-CoV-2, like SARS-CoV, depends on ACE2 (Gordon et al., 2020; Hoffmann et al., 2020; Walls et al., 2020; Zhang et al., 2020a), a type I transmembrane metallocarboxypeptidase that negatively regulates the Renin-Angiotensin system, and is expressed in the lung, kidney, and gastrointestinal tract in particular (Riordan, 2003) – all tissues shown to harbor SARS-CoV (Harmer et al., 2002; Ksiazek et al., 2003; Leung et al., 2003). Once bound, cellular entry can proceed in two ways. Firstly, evidence shows that the cellular serine protease TMPRSS2, also used by SARS-CoV, can prime the S protein extracellularly (Riordan, 2003) while the virus is bound to ACE2 (so called “shedding”), allowing for membrane fusion of the viral membrane and plasma membrane, resulting in direct viral entry at the plasma membrane. Alternatively, SARS-CoV-2 entry can be facilitated by endosomes; the S protein is primed by the pH-dependent endosomal protease cathepsin following viral uptake (Riordan, 2003), leading to fusion of the virus and endosomal membrane, and the infection. Despite the strong evidence for endocytosis as a key mechanism for CoV entry, studies have observed variable mechanisms of entry even when considering the same CoVs (Yang and Shen, 2020). This is thought to be due to the use of different cell lines, pointing to the idea that viral entry is context dependent, including both the cell type and specific virus features (Meng et al., 2020).

While entry pathways and cleavage patterns seem similar in SARS-CoV and SARS-CoV-2, some minor differences in the spike protein of SARS-CoV-2 have been noted, offering an explanation for the rapid spread of COVID-19. Both CoVs display an identical furin-like S2′ cleavage site, containing basic residues necessary for furin mediated fusion, but SARS-CoV-2 has an SPRR insertion at the S1/S2 boundary cleavage site of the S protein (Meng et al., 2020). As a result of this insertion, SARS-CoV-2 displays a significantly higher furin score than SARS-CoV (Meng et al., 2020), attributing to more efficient cleavage of the spike protein of SARS-CoV-2 that ultimately increases the viruses pathogenicity (Meng et al., 2020).

Upon successful entry into the cell, the 30 kb SARS-CoV-2 genome encodes as many as 14 open reading frames (Orfs) (Gordon et al., 2020). The genome is very similar to SARS-CoV, with both viruses containing an Orf1ab which encodes approximately 15–16 predicted non-structural proteins (Nsps) (Chen et al., 2020). At the 3′ end of the viral genome, as many as 13 Orfs are expressed from 9 predicted sub-genomic RNAs, including four structural proteins: Spike (S), Envelope (E), Membrane (M), and Nucleocapsid (N), and 9 putative accessory factors (Gordon et al., 2020). The viral genomes differ in some 3′ Orfs, where SARS-CoV-2 possesses an Orf3b and Orf10, which have limited similarities to SARS-CoV (Gordon et al., 2020). Nsps and the translated nucleocapsid remain in the cytoplasm, whereas the remaining structural proteins (S, E, and M) are translated by ER-bound ribosomes. Following translation, these structural proteins are incorporated into the ER to form virion precursors (Schoeman and Fielding, 2019). The virion precursor from the ER membrane fuse with the genomic material and the nucleocapsid in the cytoplasm, and are then trafficked out of the cell via small vesicles (Schoeman and Fielding, 2019), ultimately fusing with the plasma membrane to complete the cell cycle via exocytosis.

A recent study suggested that SARS-CoV-2 is also able to spread directly from cell to cell, avoiding the neutralizing antibodies present in the extracellular space, at a significantly higher rate than SARS-CoV (Musarrat et al., 2020). This process is mediated by expression of the S protein on cell surface, and occurs similarly to fusion that would typically occur between the viral envelope and plasma or endosomal membrane, offering an explanation for increased virulence of SARS-CoV-2 (Musarrat et al., 2020). Cell fusion leads to the development of multinucleated cells, or syncytia (Musarrat et al., 2020) whose presence is indicative of membrane fusion. Abl kinase inhibitors have been found to prevent syncytia formation, and viral infection by both SARS-CoV and MERS (Sisk et al., 2018). The Abl kinase signaling pathway is presumably involved in viral entry, predictably by interfering with actin dynamics required in virus-cell or cell-cell membrane fusion (Sisk et al., 2018).

Interactions of SARS-CoV-2 proteins have been mapped with human proteins, whereby 332 high-confidence protein-protein interactions were identified, that may lead to important targets for prevention of COVID-19 (Gordon et al., 2020). Most notably, SARS-CoV-2 interacts with multiple innate immune pathways, the host translation machinery, a Cullin ubiquitin ligase complex, and bromodomain proteins. It was observed that various Nsps target the IFN pathway, the NF-kB pathway, and two E3 ubiquitin ligases, TRIM59 and M1B1, which are known to regulate anti-viral innate signaling. This study also identified interactions between Orf6 and NUP98-RAE1, an interferon-inducible mRNA nuclear export complex. This proteome interaction of SARS-CoV affects host interferon signaling by interfering with nuclear transport (Gordon et al., 2020). It is possible that the effect is similar in SARS-CoV-2 infection as well. SARS-CoV-2 has also been observed to interact with the host translation machinery. CoV mRNAs produce proteins using cap-dependent translation, thus observed interactions between the viral proteins and the host eIF4F-cap-binding complex constituents likely play a key role in viral translation, and provide potential therapeutic targets against SARS-CoV-2. Many SARS-CoV-2 proteins are also predicted to be inserted into the ER membrane. This process in SARS-CoV is mediated by the host Sec61 translocon, and it is predicted that the process is mediated similarly in SARS-CoV-2 (Gordon et al., 2020).

SARS-CoV-2 interacts with members of a Cullin 2 (CUL2) RING E3 ligase complex (Gordon et al., 2020). This is common among viruses that tap into ubiquitin pathways to promote viral replication and pathogenesis. The Orf10 was shown to be responsible for binding to the Cullin complex, predictably hijacking it for ubiquitination and degradation of restriction factors. Finally, the SARS-CoV-2 envelope, which, as previously mentioned, resides on ER-Golgi intermediate compartment (ERGIC) and Golgi membranes, interacts with members of bromodomain and extra-terminal (BET) domain families, BRD2 and BRD4. These bromodomain proteins play a role in regulating gene transcription. A short peptide motif in the NS1 protein of an influenza A strain interferes with transcriptional processes to support antiviral response, thus it is possible that this interaction is responsible for a similar action in the case of SARS-CoV-2 as well (Gordon et al., 2020).

The largest of the accessory proteins in SARS-CoV, Orf3a, has been found to play key roles in membrane rearrangement and host cell death (Freundt et al., 2010). The 3a protein causes intracellular vesicle formation and is necessary for the Golgi fragmentation that occurs during viral infection. There is evidence that Orf3a might disrupt the function of Arf1, a Golgi regulator protein, leading to increased Golgi fragmentation (Freundt et al., 2010). Deletion of Orf3a was found to reduce host cell death after infection with SARS-CoV. It is important to note that the effects of Orf3a were investigated in SARS-CoV, but would be worth further investigation and consideration as a target for SARS-CoV-2.

Interactions between SARS-CoV and valosin-containing protein (VCP) have been observed, whereby VCP is suggested to play a role in the maturation of virus-loaded endosomes (Wong et al., 2015). VCP depletion is associated with inhibited degradation of the viral N protein, which forms a tight complex with the RNA genome in mature virions (Wong et al., 2015). This provides evidence for crucial role of VCP in the release of genetic material in the cytosol upon viral entry, and could be required for viral infection (Wong et al., 2015).

## Experience With RNAi Therapy of SARS-CoV-2

While silencing any protein is theoretically feasible using RNAi, effective target selection is essential for the efficacy of this approach. RNAi against COVID-19 disease can potentially be directed against two different categories of targets: (i) viral proteins essential in survival and replication of SARS-CoV-2, and (ii) host factors involved in cellular entry and trafficking of the virus. Below we provide a summary of various molecular targets considered for RNAi efforts, and current and past experience with the RNAi based experimental therapy.

### Host and Viral Targets for RNAi Therapy

#### Host Targets

There is increasing evidence on the importance of the endocytic pathway and the autophagy process in viral entry and replication. The components of the endocytic pathway has been suggested as important targets for development of therapeutic strategies for all species of CoV family (Yang and Shen, 2020). It is still unknown whether or not the CoV enhance the process of autophagy. In 2012, Bernasconi et al. (2012) reported a novel role for non-lipidated light-chain 3 (LC3), known as an autophagy protein, in endoplasmic reticulum-associated degradation (ERAD); CoV was suggested to hijack LC3 for replication and that silencing LC3 could inhibit viral replication based on studies performed on the mouse hepatitis virus (MHV), known as a prototype CoV. It was previously reported that MHV replication was impaired in APG5 (another component of autophagy) deficient embryonic stem cells (Prentice et al., 2004); however, in a more recent study, no significant difference was reported for SARS-CoV titers during infection of wild-type or autophagy-deficient ATG5(−/−) mouse embryonic fibroblasts (Schneider et al., 2012). The role of autophagy in internalization and replication of CoV is controversial and remains to be clarified.

The involvement of endosomes/lysosomes in internalization of CoV was first reported in 1984 for infectious bronchitis virus (IBV) and porcine epidemic diarrhea virus (PEDV) (Ducatelle and Hoorens, 1984). Since then, targets in endocytic pathway has been explored in CoV antiviral therapies. Antimalaria agents chloroquine and hydroxychloroquine have been studied for their ability to neutralize lysosomal pH and inhibit protease activity. While chloroquine was extensively studied in SARS-CoV (Keyaerts et al., 2004; Vincent et al., 2005; De Clercq, 2006) and SARS-CoV-2 (Hong, 2020; Millán-Oñate et al., 2020; Moore, 2020), hydroxychloroquine has drawn more interest as a potential treatment for SARS-CoV-2 alone (Arnold and Buckner, 2020; Yao et al., 2020; Zhou et al., 2020) or in combination with chloroquine (Fantini et al., 2020; Meo et al., 2020; Shittu and Afolami, 2020). It was recently reported that both drugs might interfere with interaction of SARS-CoV-2 with cell surface gangliosides as well, which could be additional factors in viral entry into cells (Fantini et al., 2020). However, this idea remains controversial as there are reports that show no significant difference in outcome for patients receiving hydroxychloroquine and the fact that some COVID-19 patients are already taking this medications as a prophylactic measure (Parperis, 2020).

The SARS-CoV identified in the original outbreak, and the new SARS-CoV-2 employ ACE2 as a receptor for S-protein to facilitate internalization of the virus (Prabakaran et al., 2004). ACE2 was reported to have a protective role in lungs and it gets down-regulated after SARS-CoV infection, and therefore might have a role in pathology of the virus as well (Kuba et al., 2006). A small clinical study in Wuhan on critically ill patients showed a worse outcome in patients with hypertension and diabetes mellitus, which was speculated to be due to overexpression of ACE2 receptor in alveolar epithelial cells (Rico-Mesa et al., 2020). On the other hand, it has been shown that soluble ACE2 has a protective role for many organs including lungs so that recombinant ACE2 was also suggested as a therapeutic strategy (Rossi et al., 2020). Proteolytic cleavage of the ectodomain of ACE2 is performed by 2 different proteases: ACE2 cleaved by TMPRSS2 enhances internalization of SARS-CoV-2, while Tumor Necrosis Factor-Alpha Convertase (ADAM17)-cleaved ACE2 offers protection to organs, including lungs (Xiao et al., 2020). Overall, this seemingly paradoxical roles for ACE2 and the possibility of over-expression of ACE2 as a result of therapy with ACE inhibitors have ignited discussions over the benefits and potential risks of targeting ACE2 as a therapeutic strategy in COVID-19.

#### Viral Targets

Instead of host targets, targeting viral proteins might be a more direct (specific) and efficacious approach. In addition to the open reading frames, the RNA genome contains sections responsible for expression of the four proteins (S, E, M, and N proteins) that can act as RNAi targets (Phan, 2020; Pillay, 2020). S-glycoprotein received most attention as it plays a major role in cell entry via ACE2 receptor and antibody binding (Walls et al., 2020). It is the receptor binding domain (RBD) of S-glycoprotein that interacts with the peptidase domain of human ACE2 (Li F. et al., 2005). S-glycoprotein also contains a fusion domain and a transmembrane domain. Binding of the S-glycoprotein to ACE2 exposes the cleavage sites of the protein to cellular proteases. Cleavage is performed by transmembrane protease serine 2 and other cellular proteases and it triggers fusion and endocytosis (Pillay, 2020). S-glycoprotein is a 150 kDa, highly N-glycosylated with similarities to the structure of S-protein in SARS-CoV; out of 14 aa residues in RBD region, only eight are strictly conserved in SARS-CoV-2. As mentioned, enhanced spreading efficiency observed in SARS-CoV-2 compared to other β-CoVs could be attributed to enhanced activity of furin-like cleavage site in SARS-CoV-2 that facilitates S-protein priming (Rabaan et al., 2020). The subunit vaccines developed for MERS-CoV and SARS-CoV (not approved for use in humans) are based on full-length S-protein, RBD, non-RBD S-protein fragments, and non-S structural proteins (Wang et al., 2020). Targeting S-glycoprotein synthesis in host cells via RNAi could potentially reduce the availability of this protein in host cells for viral assembly, leading to sub-optimal assembly of the virus and reduced infectivity.

In addition to S-protein, E-, and M-proteins are structural proteins involved in the formation of viral coat (Wu et al., 2020). The genome domain responsible for expression of E-protein is well-conserved. In fact, in a study on mutations among 68 samples of SARS-CoV-2, which identified 42 missense mutations in all the major non-structural and structural proteins, none was detected in E-protein (Phan, 2020). The E-protein is an 8–12 kDa protein and plays a critical role in the virus assembly and release. It is also involved in ion channel activity that is required for pathogenesis of SARS-CoV, and possibly SARS-CoV-2 (Rabaan et al., 2020). The N- and C-terminal of M-protein are ectodomain and endodomain, respectively. It is found in virion as a dimer and is involved in maintaining the viral membrane curvature and binding to nucleocapsids (Neuman et al., 2011). The N-protein is incorporated into nucleocapsid and each domain can bind to RNA (Hurst et al., 2009) via its phosphorylated residues.

### Industry Focus on RNAi Therapy of CoV

To assess the potential of RNAi in management of COVID-19, one can inspect the response of pharmaceutical companies focused on development of RNAi mediated therapies. In the short time following the publication of SARS-CoV-2 genome, proprietary designs were implemented by several companies to identify effective siRNAs and explore the possibility of such siRNAs for prevention and treatment of SARS-CoV-2 infections. We are aware of this activity through company press releases since there has not been any time to complete peer-reviewed studies on silencer designs and efficacy at the time this review was written. However, the press releases do provide an indication for the prospect of RNAi approach. Vir Biotechnology (San Francisco, United States) and Alnylam Pharmaceuticals (Boston, United States) have reported a joint activity to explore a library of siRNAs in this disease (Alnylam Pharmaceuticals, 2020). Alnylam reportedly designed and synthesized over 350 siRNAs targeting all available SARS-CoV and SARS-CoV-2 genomes, including targets in highly conserved regions of the coronavirus RNAs, presumably due to their better retention in viral progeny (i.e., stable target) and reduced chance of “inactivating” mutations. The OilX Pharmaceuticals (Suwan, South Korea) is also pursuing siRNAs that target highly conserved regions of coronavirus RNA (OliX Pharmaceuticals, 2020). Patent filing was reportedly undertaken that provide broad composition of matter claims to more than 30 siRNA designs that target the genome of the COVID virus with highly conserved regions among CoVs. Specifically, the targets are selected among the proteins that play important roles in virus replication such as 3CL-protease, RNA-dependent, RNA polymerase, and S-protein. Sirnaomics (Gaithersburg, United States) (NS Healthcare, 2020) has also identified potent siRNAs, which target the crucial genes for CoV infection and replication. All three companies are interested in inhalational delivery of the siRNA formulations and a variety of device configurations are likely to be implemented for inhalational delivery. The relative potencies of the chosen siRNAs and therapeutic efficacy will be better assessed once peer-reviewed outcomes are reported in the literature.

### Past Experience With siRNA and shRNA Therapy of CoV

In early studies that utilized siRNA against SARS-CoV (Hong Kong strain), among the seven sequences used to target various regions, the most effective ones were two siRNAs (out of three) directed against the S-protein (Wu et al., 2005). As in the studies that aimed to inhibit viral binding and explored effective vaccine epitopes, silencing siRNAs against S-proteins seems most effective with 85–90% reduction in viral load as assessed by PCR analysis. An siRNA against the Leader sequence was relatively less effective (∼50%, estimated) in that study (Wu et al., 2005). The emergence of the S-protein as a therapeutic target was also independently verified (Qin et al., 2004; Zhang et al., 2004) with shRNAs, where one study employed SARS-CoV infection directly (using strain BJ01).

A separate study focused on a Leader sequence in SARS-CoV (clone BJ01) that was predicted to be common to all CoV and expected to undergo minimal mutagenesis, providing a more stable target for silencing (Wang et al., 2004; Li T. et al., 2005). This sequence also appeared in the S-protein. An shRNA from a pDNA expression system was designed against this target whose delivery ahead of SARS-CoV transfection reduced the viral load. This study, however, suggested superior effects with targeting Leader sequence as compared to S-protein gene unlike the previous cases (Zhang et al., 2004; Wu et al., 2005). Differences in the silencing efficiencies related to targeting different regions of the genome as well as the RNAi mode used (i.e., siRNA vs shRNA) might explain the differences in these results. A study by Ni et al. (2005) also focused on a non-structural protein 1(NSP1), derived from the 5′ leader end of the genome. When shRNA transfected cells were challenged with the SARS-CoV, there was significant protection of the cells, with PCR based reduction of virus load being 80-500-fold lower.

Beyond the 5′ Leader sequences and S-protein gene, targeting the gene coding for the nucleocaspid N-protein was also explored with effective reduction of viral loads using the shRNA approach (Tao et al., 2005; Zhao et al., 2005). It was possible to silence the N protein in an intramuscular mouse injection model as well using shRNA approach (Zhao et al., 2005). Recent evidence indicates that N-protein may act as a viral suppressor of RNAi mechanism in host cells (Cui et al., 2015), so that its inhibition could enhance host response indirectly. N-protein was also targeted by Cao et al. (2011) that utilized 16 isolates of SARS-CoV to choose 3 regions that were well conserved in the N-genes, providing a better chance of a universal target. The silencing was implemented with shRNA in robust 293T cells. It was noteworthy that suppression of N-gene also resulted in increased INF-β secretion, providing an additional mechanism to fight the virus.

Lu et al. (2004), Meng et al. (2006), and He et al. (2003) chose to target highly conserved RNA-dependent RNA polymerase (RDRP) gene, one of the genes that is conserved in different strains of CoV, and showed effective silencing of this gene expression using siRNA and shRNA. There was no synergistic activity when effective siRNAs were combined in the study of He et al., 2003, perhaps due to the choice of a single gene locus for targeting that is not conducive for synergistic activities (i.e., targeting multiple genes might have allowed synergism). However, the study noted differential sensitivity of the RDRP gene to different siRNA sequences, emphasizing the importance of siRNA design even for individual targets. Viral envelope E-protein was also targeted with siRNAs (Meng et al., 2006), but RDRP appeared to be more effective target in reducing viral replication. It was worthwhile to note that within the RDRP, widely different efficiencies were noted for several siRNAs targeting different regions of the gene. The latter was attributed to better assembly of RISC complex or stability of siRNAs inside the cells.

M-protein was another target that was explored (Qin et al., 2007), where the specificity of designed siRNAs were validated, but no SARS-CoV infection and/or its inhibition was investigated in that study. A Chinese patent subsequently claimed pharmaceutically useful formulation with specific siRNA sequences against M-protein as the basis of a therapy (Ying et al., 2006). In a more sophisticated study, M-protein gene from 15 SARS-CoV isolates were compared to identify relatively stable regions and two specific shRNAs were explored for silencing. Consistent with previous studies (Qin et al., 2007), targeting the 3′ portion of M-gene was more effective in reducing the target mRNA levels. M-protein is also an inhibitor of the master-regulator nuclear factor kappa B (NF-kB) and its signaling pathway, which is closely associated with regulation of inflammatory cytokine secretion. It was not surprising to see a reduction in INF-β secretion after M-protein silencing (Ying et al., 2006). The reduction of M-protein synthesis may have indirect effect in reducing the detrimental inflammatory reaction.

Several accessory proteins with no significant sequence homology to viral proteins of other CoVs were noted in SARS-CoV. These proteins derived from subgenomic RNAs were also effective targets, as effective as the commonly targeted S-protein (Akerström et al., 2007). It was interesting to note that two or more mis-match pairing was detrimental for the silencing activity of the silencer molecules, whereby genomic mRNA with such base mismatch siRNAs were not silenced. This observation has important implications for the mutated forms of the virus.

In the most advanced study involving testing on primates (Rhesus macaque), Li B.-J. et al. (2005) and Tang et al. (2008), from a new set of 48 distinct siRNAs targeting regions of entire SARS-CoV genome, identified two leading siRNAs (against S-protein and ORF1b regions) and delivered them as a mixture to SARS-CoV-infected fatal Rhesus monkey kidney (FRhK-4) cells; the siRNAs displayed potent and synergistic activities. Intratracheal administration of siRNAs were accomplished in aqueous buffers, D5W (5% dextrose) and Infasurf solution (i.e., extract of natural surfactant from calf lungs containing phospholipids, neutral lipids, and hydrophobic surfactant-associated proteins B in 0.9% aqueous NaCl) along with a reporter gene to asses silencing efficiency. No special carrier was needed to implement RNAi. Using the simpler buffer D5W and a Rhesus macaque model that displayed features typical of human SARS, intranasal siRNA delivery resulted in effective lowering of symptoms (based on temperature measurements) in a prophylactic, co-delivery (with CoV) and postexposure groups, as well as reduced histopathological changes in the lung tissue of the treated animals. The replication of the virus was halted (based on qPCR analysis) typically in three out of four treated primates. The study provided some guidance about the clinical dose that may be required with siRNA administration (∼10 mg/kg) but that is likely to depend on the details of the siRNA formulation and whether a carrier will be employed along with siRNA.

### Critical Issues in Delivery of RNAi Agents

The silencing studies described so far typically employed liposomal commercial carriers [e.g., Lipofectamine (Wu et al., 2005), FuGene (Meng et al., 2006) and older generation lipofection reagents] that are not intended for further animal studies. Others have used the classical Ca/P mediated transfection (Wang et al., 2004; Li T. et al., 2005) whose utility for animal models is not known. No practical carrier emerged in these studies suitable for clinical applications since most were focused on demonstrating the target validation with little emphasis on clinical translation. The cell models used in these studies were also easy-to-transfect cells such as 293T and Vero cells, whose features are far different from the primary cells intended for modification. Without the use of primary cells and animal models that better represent the clinical scenario, delivery issues to be faced by RNAi agents in clinical setting will not be solved. To implement RNAi, most studies concentrated on the shRNA, where the short interfering RNAs are derived from plasmid-based expression systems, due to practical aspects of conducting the initial proof-of-principle studies. This system is convenient to implement (especially in easy-to-transfect cell lines), more economical and allows stable expression of interfering RNA sequences. However, it is unlikely for the shRNA approach to be employed as a therapy. Most of these studies also focused on “prevention of infection” whereby the RNAi agents are induced first and then viral infection is attempted. It is more likely to demonstrate an efficacy in this kind of a set-up but the clinical reality is the reverse; the patients are already infected with the CoV before the RNAi agents need to be administered. Although one can envision a prophylactic use of RNAi with stably integrated shRNA expression systems (if such a system can be made to turn on gene expression “on demand”), this will probably not be a clinical reality. One needs to deploy shRNA or siRNA after the infection and retain the viral load at manageable levels for the immune system to manage the disease.

It was intriguing to see effective siRNA delivery in a primate model (Li B.-J. et al., 2005; Tang et al., 2008) even without the use of a carrier. Our (and others’) extensive cell culture experience indicates a lack efficacy by naked unmodified siRNA sequences in the absence of carriers, yet this primate study observed good anti-viral efficacy without a carrier. The authors in that study noted that the use of polyethylenimine (PEI) was associated with lung inflammation in the employed mouse model, clearly emphasizing the need for a more biocompatible system in delivery. If a biocompatible carrier could be identified, it is likely that the effective dose could be reduced below 10 mg/kg, to more acceptable levels as ∼1 mg/kg that was suggested for systemic injections. Without a carrier, some degree of degradation is bound to happen (half-life of free siRNA in serum is generally regarded to be <30 min) so that a “protected” siRNA should have increased potency. It is likely that the administered siRNA is “actively carried” into pulmonary cells by non-specific pinocytosis or macrocytosis. Alternatively, local biomolecules may complex and present the siRNA for cellular uptake reminiscent of synthetic carriers. The major soluble extracellular macromolecules in lungs (e.g., hyaluronic acid, SPARC) are anionic and are not likely to interact with anionic siRNA to facilitate uptake. However, with >300 members in the proteome of lung tissue (Burgstaller et al., 2017), complexing proteins are bound to be present, even if only cationic domains participate in siRNA sequestration and cell presentation. Lung surfactants are another possibility for facilitating siRNA delivery into the cells (Guagliardo et al., 2018; Autilio and Pérez-Gil, 2019). The surfactants having compositional structure of lipids (∼90%) and proteins (∼10%) might entrap siRNA with cationic domains and enhance membrane crossing into the cells due to lipid components. Our studies on synthetic lipophilic carriers (Incani et al., 2010) showed that these two domains could be engineered to obtain effective siRNA delivery agents to a variety of cells. It may be possible to set-up ECM mimics of pulmonary tissue (Evans and Lee, 2020) to better understand the mediators of siRNA delivery into the cells, given the implication of this process on the potency of siRNAs agents for anti-viral activity.

It is likely that there is no “magic” viral target for most potent intervention, and several alternative targets might present itself for silencing. Among the genes encoding for E-, M-, and N-proteins, a library of 26 siRNA effectively reduced all three target proteins (albeit at different levels), but siRNAs with equipotency could be demonstrated against three separate genes (Yi et al., 2005). It was possible to improve the potency of less effective siRNAs by enhancing the internal instability at the 5′-antisense terminal base pair (by adding mis-match pairs) but this was not attempted to improve the potency even further.

Significant efforts will be required to identify exceptionally effective RNAi therapy, not only to demonstrate a robust response but to justify the relatively higher cost of the therapy as compared to possibly emerging “re-positioned” drugs. The latter is expected to be <$1,000 per treatment based on our estimates while the RNAi agents are likely cost significantly more due to the need for developing a sophisticated (costly) biomolecule as a drug with an associated delivery system. The potency is likely to depend on the choice of targeted gene *a priori*, followed by specific sequences employed to silence that particular gene. The resistance development is also likely to depend on similar factors; the targets that are less amenable to mutation should be chosen to prevent resistance development and allow siRNA use among the most patients (given the more likelihood of finding CoV with similar genomic parts among the population). Combinational delivery is likely to play a significant role in potency (KC et al., 2017). Given the similarity in the physicochemical features of individual siRNA molecules, it will not be a significant challenge to deliver a combination of two or more siRNA molecules at the same time. This is unlike the conventional drugs that display different physicochemical features due to smaller size and will likely require separate administration in a patient. The choice of the combination(s) will be important, and a critical issue will be whether to target the same gene with multiple siRNAs or to employ different sets of genes for a comprehensive assault on the CoV. In this respect, He et al. (2006) explored siRNA against the full spectrum of viral proteins, where reduction of 67–83% viral load could be obtained with specific siRNAs against S-, N-, M-, and E-proteins. More importantly, various combinations of siRNA targeting different regions of the genome was synergistically effective to stop viral replication with greater potency (Table 1).

**TABLE 1:** Desirable features of siRNA agents for SARS-CoV-2 therapy.

• Potent antiviral response at <100 nM in cell culture and 1–10 mg/kg dose in animal models. • Active after inhalational delivery. • No homology with the human genome. • Minimally mutating targets in CoV genome and/or common genome sequences among CoV family members. • siRNAs with synergistic activity.

## CoV Infection and Immune System: Focus on Cytokine Storm

Some of the early studies that noted linkages between specific SARS-CoV proteins and their role in modulating cytokine release in cell culture were pointed out earlier. Additionally, M-protein of SARS-CoV was shown to inhibit NF-kB expression (in addition to Cox-2) that may contribute to SARS pathogenesis (Fang et al., 2007). Early production of cytokines and chemokines in lungs primes the tissue for influx of NK cells, macrophages, and plasmacytoid dendritic cells (pDC). Such an influx can lead to a second wave of distinct cytokine and chemokine production that can further dilate the vasculature and cause pneumonitis of the lungs (Chen et al., 2010). Early studies with SARS-CoV-infected dendritic cells showed low expression of anti-viral cytokines IFN-α/β/γ and IL-12p40, moderate up-regulation of proinflammatory cytokines TNF-α and IL-6, but significant up-regulation of inflammatory chemokines MIP-1α, RANTES, IP-10 and MCP-1 (Law et al., 2005). In macrophages, SARS-CoV failed to induce anti-viral IFN-α/β gene expression as well (Cheung et al., 2005). The lack of anti-viral cytokine response despite chemokine up-regulation could facilitate immune evasion by the SARS-CoV. Airway and lung memory CD4(+) T cells were critical in the initial response to SARS-CoV (Zhao et al., 2016). Interfering with key cytokines could be a fruitful approach to minimize life-threatening tissue damage in the lungs. Given the spectrum of altered cytokines, interference with “master” regulators might be more fruitful rather than late effectors. IL-17 might be a feasible target in this regard given its central role in induction of chemokines. In the mouse model deficient for IL-17RA, viral infection was shown to reduce the immune cell migration to the lungs, leading to lower morbidity in the animals. The pro-inflammatory cytokines TNF-α, IL-1β, and IL-6 were attenuated in IL-17RA deficient mouse, fulfilling the role of master regulation in this regard. IL-17 was also highly up-regulated in healthy PBMC infected *in vitro* with SARS-CoV (Ng et al., 2004) and one can envision targeting IL-17 with RNAi agents to attenuate the cytokine storm and minimize the inadvertent damage. This may be an alternative to antibody therapy against IL-17 being commercially pursued by Novartis.

One study noted that expression of cytokine genes was completely absent in PBMC isolated from SARS-CoV patients, and the immune-related genes which were over-expressed were usually associated with innate-immune response against bacterial infection and not viral infection (Reghunathan et al., 2005). However, this may not reflect the local tissue response. The PBMCs from healthy individuals did respond to SARS-CoV infection by early (12 h) cytokine response, including the involvement of the master regulator NF-kB (Ng et al., 2004) in the absence of pro-inflammatory cytokines TNF-α, IFN-γ, and IL-6. As in SARS-CoV infections (Jiang et al., 2005), the systemic levels of pro-inflammatory cytokine IL-6 was particularly associated with the severity of SARS-CoV-2 pathology (Zhu et al., 2020) and anti-IL-6 antibody therapy seems promising in early studies exploring its utility in the therapy SARS-CoV-2 (Xu et al., 2020). IL-6 might serve as another target of RNAi agents but whether targeting a single cytokine among several pro-inflammatory cytokines will be efficacious enough remains to be seen. Another chemokine, Interferon-inducible protein-10 (IP-10; also known as CXCL10) detected in SARS-CoV infections (Jiang et al., 2005) might be critical since its expression levels was shown to be associated with disease severity in SARS-CoV-2 patients (Yang et al., 2020).

The increased mortality among elderly SARS-CoV-2 patients is well established, whose underpinnings is important to understand in order to develop an effective therapy for this patient population. In a primate (cynomolgus macaques) study comparing gene expression profiles in aged vs young subjects from lung tissues, the aged subjects displayed higher gene expressions associated with immune and inflammatory responses (Wu et al., 2005), while the central transcription factor NF-kB playing a major role in this response. Two additional cytokines differentially expressed were IL-8 (up-regulated), as in PBMC (Jiang et al., 2005), and IFN-β (down-regulated) in aged primates. IFN-β supplementation attenuated the excess pro-inflammatory response in aged primates and reduced the disease severity. This improvement was achieved without changes in viral load of subjects (Wu et al., 2005), suggesting that addressing viral load alone might not be absolutely necessary. It might be possible to augment local anti-viral (with IFN-β like agents) response without altering the pro-inflammatory processes. Similar observations were noted in a mouse model, where adult Balb/c mouse displayed a greater cytokine response to SARS-CoV infection compared to young mice (Zhu and Qu, 2009). Endonuclease (EndoU) region incorporated in the non-structural protein 15 (nsp15) domain of CoV, seems critical to suppress the initial IFN-β response (Kindler et al., 2017), and RNAi agents targeting this region might suppress viral load while inhibiting the viral attempts to dampen the host anti-viral response.

A generic approach to control cytokine storm is to employ corticosteroids. Recent evidence from a meta analysis indicated that corticosteroid use was associated with delayed virus clearing, no significant reduction in deaths, prolonged hospitalization and use of mechanical ventilation increased (Li et al., 2020). The efficacy of the non-steroidal anti-inflammatory drugs, the inhibitors of cyclooxygenase (COX) 1 and 2, are also not known even though indomethacin was shown to reduce SARS-CoV replication in dogs (Amici et al., 2006). S-protein in SARS-CoV could activate the inducible COX-2 (Liu et al., 2007) but whether COX-2 inhibition results in an effective therapy remains to be determined. Perhaps more specific interference with the specific mediators of cytokine storm might provide a more desirable outcome, but this remains to be seen.

## Inhalational Delivery

Inhalation delivery of drugs and vaccines, both oral, and nasal, has been introduced as an alternative to conventional delivery approaches with many advantages including ease of administration and rapid onset of action. Pulmonary delivery has been used for localized and systemic delivery of various drugs such as peptides, proteins, DNAses and vaccines (Bhavane et al., 2003; Velasquez et al., 2011). Live attenuated influenza vaccines, which have been available as inactivated form since 1940s through intramuscular, was introduced in US in the form of nasal spray in 2003. More attention has been recently given to nasal inhalations of vaccines since this route can provide better mucosal immune responses (Fiore et al., 2009). The mucosal immunity is critical in providing protection against pathogens localized in mucus membranes as a result of entry through nose or mouth. The immunity from localized formulation better encounter and neutralize the pathogens at the point of entry before they enter the systemic circulation (Yusuf and Kett, 2017). Nasal formulations in the market are mainly in the form of drops, liquid sprays, powder sprays or gels. Drops are the most common formulations and require either tilting back the head or laying down to prevent leaking out and to ensure that the droplets of liquid remain in the nasal cavity. This could be challenging in animal studies and large human vaccination settings. Gels and sprays could overcome this issue since the gelling and mucoadhesive agents in the formulations would prevent leaking out of nostrils. Powder formulations could provide more stability compared to liquid formulations, and may be delivered deeper in the nasal cavity (Yusuf and Kett, 2017). Nanoparticle formulations have recently garnered more attention for their ability to ability to deliver a range of therapeutic agents, including nucleic acids and RNAi agents.

Inhalational delivery of RNAi agents and dry powder formulations of siRNA was recently reviewed (Dua et al., 2019; Keil and Merkel, 2019). In 2020, Fukushige et al. (2020) described hyaluronic acid coated liposomes as spray freeze-dried nanoparticles for pulmonary delivery of siRNA in lung cancer as a superior alternative to non-modified liposomes. Intranasal inhalation of a formulation comprising of ovalbumin and an immunoadjuvant loaded in poly(lactic-co-glycolic acid) (PLGA) nanoparticles induced more robust antigen-specific CD8 + T-cell responses in comparison to intraperitoneal administration of the same formulation (Li B. et al., 2016). Chitosan is another polymer used extensively in inhalation therapy due to its mucoadhesive properties either as the carrier or coated on the surface of other nanoparticles. Chitosan was used to encapsulate the influenza vaccine in dry powder formulations for inhalation therapy. The result supported the appropriateness of the dry powder chitosan nanoparticles for nasal delivery owing to the mucoadhessive property of chitosan and the nano size range of the formulation (Muralidharan et al., 2015). The pulmonary surfactant Curosurf-coated on nanoparticles was also used to enhance the inhalation delivery siRNA; SP coating enhanced the nanoparticle uptake by alveolar macrophages, which are the main targets in the treatment of inflammatory pulmonary diseases (Merckx et al., 2018). Finally, we note that a recent study showed that siRNA inhalation alone (without a carrier) appeared to be as effective as siRNA formulated with the traditional polymeric carrier PEI, perhaps suggesting the unique features of inhalational route to sustain “free” siRNA activity (Ito et al., 2019).

## Future Perspectives

**FIGURE 3:**
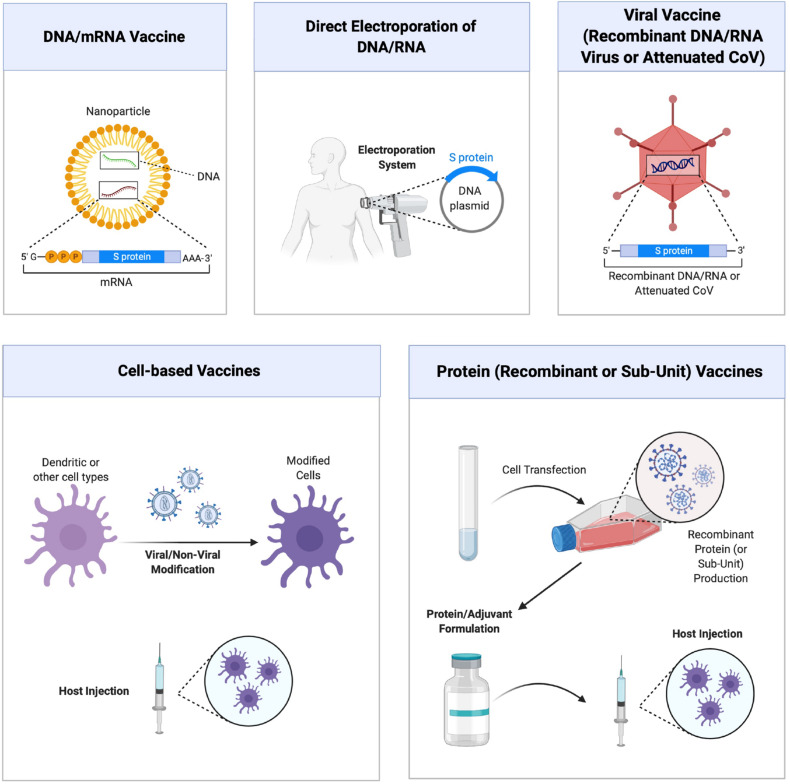
Different approaches to vaccine development against COVID-19 disease. Main strategies are schematically shown, which relies on (from top left, clockwise) (i) nucleic acid (DNA and RNA) vaccines delivered with nanoparticulate carriers and coding for specific viral sub-units or virus-neutralizing agents, (ii) direct administration of DNA/RNA expression systems with forced expression, (iii) viral vaccines composed of recombinant or attenuated viruses, (iv) vaccines derived from recombinant viral proteins or purified sub-unit proteins, and (v) cell based vaccines relying of modification and administration of cells. Figure courtesy of BioRender.

The rapidly changing literature on SARS-Co-2 makes it difficult and, at the same time exciting, to predict future developments on the use of RNAi agents for managing the COVID-19 disease. It is generally agreed that effective COVID-19 vaccines will be a permanent solution to viral infections and numerous strategies are developed to this end (Figure 3). It is likely that more than one strategy could be successful to this end, some strategies might be more suitable for certain patient populations and that the nature of viral evolution might hamper effective vaccine development efforts (for reviews on COVID-19 vaccine, see Conte et al., 2020; Pandey et al., 2020; Wang et al., 2020). It is also likely that the immunizations might not be permanent and that some individuals could not develop the required immunization at all. Drug therapies and therapies based on RNAi could be an alternative in less than desired vaccination outcomes. At the time of writing of this manuscript, no therapeutic RNAi studies were reported on SARS-CoV-2 silencing, so that we relied on past experience with similar CoV infections to shed light on critical issues and fruitful avenues that may be possible in the future. Potential siRNA sequences against SARS-CoV-2 genome are beginning to be reported in the literature (Chen et al., 2020), but their validation remains to be tested. Besides the role of known mediators summarized above, SARS-CoV encodes numerous accessory proteins whose importance in natural infection process is currently unclear (Chen and Zhong, 2020). Some of these proteins could be used as additional targets for RNAi mediated silencing, but their importance remains to be explored. Table 2 summarizes some of the CoV targets whose importance as RNAi target remains to be explored. Beyond the viral targets, host factors might also provide opportunities for RNAi agents. It is likely that targeting host factors might be more likely to lead to undesired effects from RNAi agents, but they could provide alternative targets in the fight against SARS-CoV-2. Mechanistic insights as outlined in section “Mechanism of Cell Entry and Infection for SARS-CoV-2” can provide individual targets worthwhile to pursue, but a more fruitful approach could be deliberate (non-biased) RNAi screens that could rank the importance of various targets and yield leads with relative ranking of their efficiency. Studies toward this goal, a common approach in anti-cancer therapies, was recently reported with SARS-CoV and identified dozens of promising host factors for silencing and reduction of viral load (Dirmeier et al., 2020). Similar lines of enquiry with SARS-CoV-2 remains to be reported and it will be so important to identify if the critical host factors are common in the case of both types of viruses. In this case, our confidence to translate the know-how generated from the SARS-CoV to the SARS-CoV-2 will be greatly enhanced.

**TABLE 2:** Other potential targets (from SARS-CoV) for silencing and their perceived function.

• ORF-9b: Mitochondrial manipulation to limit IFN response (Shi et al., 2014). • Papain-like Protease (PLpro): Up-regulation of TGF-β1 mediated pro-fibrotic responses (Li S.-W. et al., 2016). • ORF-3a, ORF-4a: May function as ion channel that may promote virus release (Lu et al., 2006; Zhang et al., 2014). • Non-structural Protein 15 (nsp15): Anti-apoptotic function by inhibiting MAVS-induced apoptosis (Lei et al., 2009). • Viral 7a Accessory Protein: Suppression of host silencing (Karjee et al., 2010). • Protein 6: Blocks nuclear import of macromolecules from cytoplasm (Hussain et al., 2008).

Past investigations on non-coding microRNAs (miRs) have opened up new possibilities particularly into development of malignancies and their therapy, but this remains a vastly understudied area in the case of SARS-CoV. The significant changes in endogenous miR profiles of cells transfected with porcine hemagglutinating encephalomyelitis virus (HEV), for example, have been noted (Fan et al., 2020; Hu et al., 2020). miR-1246 was recently linked to regulation of ACE2 expression in airway epithelium (Zhang et al., 2020b) and several miRs have been predicted to bind to the SARS-CoV-2 genome (Chen and Zhong, 2020). Such miRs could serve as RNAi targets and/or might be deployed directly (either as miR mimics or anti-miRs), or indirectly as a result of modulation with pharmacological agents. Identifying the pertinent miRs and revealing their mechanistic involvement is bound to provide effective leads not predicted before, such as the case of Syndecan 1 involvement, which is regulated by miR-10a-5p, and whose mimic significantly altered the course of HEV replication (Hu et al., 2020).

While the scientific rationale may identify optimal targets, intellectual property considerations might as well determine the target choice for individual pharma companies to better navigate the drug development process without infringement. If cancer experience was to serve as a guide in this effort, we think that targeting alternative genome regions might be more fruitful especially if CoV might get to heavily rely on certain biomolecules for replication in the face of drug assault, which might be its Achilles heel. We, and others, observed that cultivating transformed cells in the presence of drugs allow rapid resistance development, but the cells become “too” reliant on certain mediators, such as anti-apoptotic proteins, whose silencing with RNAi makes them exceptionally sensitive to conventional drug and/or siRNA treatments (Aliabadi et al., 2013).

Finally, one must consider the safety of siRNAs in reaching the desired treatment target. It is possible to design CoV-specific siRNAs with no “theoretical” cross-reactivity to human genome, but this issue remains to be validated. Partial homology and resulting hybridization to non-target mRNAs might lead to toxic effects so that potency vs safety issues might have to be weighted in choosing the final formulation. Inhalational delivery is bound to aid in minimizing the safety concerns, since less drug doses might have to be delivered to the critical site of viral infection (i.e., lungs). Respiratory complications due to deposition of a foreign material in the airways is always a concern but the benefits of direct inhalational delivery are likely to overweight its shortcomings. At the present time, no inhalational delivery of RNAi agents against CoV have been reported, but this is bound to change in the near future. Various technologies seem to be in place for dry powder and liquid formulations, as well as mucoadhesive formulations for this end, and siRNA and pDNA (to code for shRNA) are now being incorporated into such formulations with functionally active form. Different lines of attack are anticipated against SARS-CoV-2 in the near future with different degree of success.

## Author Contributions

HU conceptialized, drafted, and edited the manuscript. KP drafted specific sections, generated figures, and edited the manuscript. HA and AH conceptualized and drafted specific sections of the manuscript. All authors contributed to the article and approved the submitted version.

## Conflict of Interest

HU is a founder and share holder and KP is an employee of RJH Biosciences Inc. that commercializes delivery systems for nucleic acids. The remaining authors declare that the research was conducted in the absence of any commercial or financial relationships that could be construed as a potential conflict of interest.
